# Holistic View of Starch Chemistry, Structure and Functionality in Dry Heat-Treated Whole Wheat Kernels and Flour

**DOI:** 10.3390/foods11020207

**Published:** 2022-01-12

**Authors:** Jana van Rooyen, Senay Simsek, Samson Adeoye Oyeyinka, Marena Manley

**Affiliations:** 1 Department of Food Science, Stellenbosch University, Private Bag X1, Matieland 7602, South Africa; 20783353@sun.ac.za; 2 Department of Food Science, Purdue University, West Lafayette, IN 47907, USA; ssimsek@purdue.edu; 3 Department of Nutritional Sciences, Faculty of Health and Medical Sciences, University of Surrey, Guildford GU2 7XH, UK; s.oyeyinka@surrey.ac.uk or; 4 Department of Biotechnology and Food Technology, University of Johannesburg, Johannesburg 2001, South Africa

**Keywords:** thermal treatment, wheat grains, starch damage, crystallinity, pasting properties, retrogradation

## Abstract

Heat treatment is used as a pre-processing step to beneficially change the starch properties of wheat flour to enhance its utilisation in the food industry. Heat-treated wheat flour may provide improved eating qualities in final wheat-based products since flour properties predominantly determine the texture and mouthfeel. Dry heat treatment of wheat kernels or milled wheat products involves heat transfer through means of air, a fluidising medium, or radiation—often resulting in moisture loss. Heat treatment leads to changes in the chemical, structural and functional properties of starch in wheat flour by inducing starch damage, altering its molecular order (which influences its crystallinity), pasting properties as well as its retrogradation and staling behaviour. Heat treatment also induces changes in gluten proteins, which may alter the rheological properties of wheat flour. Understanding the relationship between heat transfer, the thermal properties of wheat and the functionality of the resultant flour is of critical importance to obtain the desired extent of alteration of wheat starch properties and enhanced utilisation of the flour. This review paper introduces dry heat treatment methods followed by a critical review of the latest published research on heat-induced changes observed in wheat flour starch chemistry, structure and functionality.

## 1. Introduction

Wheat (*Triticum aestivum* L.) is a staple crop that provides energy, proteins, dietary fibre, vitamins, minerals and phytochemicals that are essential dietary components for many individuals daily [1,2]. It is the only cereal that has the potential to form a dough, entrap air bubbles during mixing and retain its gluten structure once baked. These properties are essential for breadmaking. Starch, representing approximately 85% (dry basis) of flour, acts as the structural element during baking [3,4]. During mixing and baking, starch contributes to water absorption, gelatinisation and pasting behaviours which are responsible for the textural properties of the crumb and crust. Upon cooling and storage, starch recrystallisation results in the staling of baked products [5]. With the ever-growing population and consumer demand, it is necessary to ensure the final products are of high quality—each with its specific characteristics and expected shelf life.

A number of studies have recently investigated thermal techniques as pre-processing methods, applied to whole wheat kernels or flour, to improve the properties of wheat flour and enhance its utilisation in breadmaking [6,7,8]. This necessitates the investigation of the impact of thermal treatment on wheat flour characteristics, such as starch damage and pasting properties as well as bread staling, due to heat-induced changes in starch properties.

Several promising non-thermal technologies, i.e., ozone, ultrasound, cold plasma, pulsed electric field, high-pressure and high homogenisation processing have recently emerged [9]. These technologies have, however, only been investigated for starch modifications of isolated starches and not for whole wheat kernels or flour. 

Thermal treatment of wheat adds value to the final product quality by contributing to flavour, aroma, palatability and storage qualities [10]. Modification of wheat flour properties may provide improved organoleptic properties in final wheat-based products, as flour properties predominantly determine the texture and mouthfeel [2]. Physical modification may improve its functional properties [4], e.g., improved cooking performances in noodles and the sensory qualities of cakes [2,5]. Heat treatment enhances the digestibility of wheat due to physical modifications, i.e., protein denaturation and starch gelatinisation [6,11,12]. Wheat bran contains significant amounts of vitamins, minerals, fibres and antioxidants including carotenoids, phenolic acids and tocopherol [13]. Consumption of these compounds reduces inflammation and contributes to the prevention of heart diseases and cancer. However, these compounds have low bioavailability, as they are entrapped in the strong cell wall structure [14]. Heat treatment of wheat grains is a promising pre-processing treatment technique to improve the bioavailability of such compounds [15]. It also reduces the microbial load, mycotoxin concentration and enzymatic activity by lowering the water activity, hence ensuring an extended shelf life [12,16,17]. 

Thermal treatments used in the wheat industry can be divided into two main categories subject to the addition of water during treatment, namely dry and hydrothermal [4]. Dry heat treatment may modify structural changes in gluten proteins and starch granules, resulting in changes in rheological properties, viscosity, water holding capacity and the rate of retrogradation [4]. Hydrothermal treatment, on the other hand, has been used to modify flour to act as thickening agents. The water introduced during treatment pre-gelatinises the starch and allows for the molecules to attain greater mobility, which consequently denatures wheat proteins, preventing the formation of a gluten network [4,18]. 

This review focuses on dry thermal treatment only and specifically on its use as a pre-processing method for whole wheat kernels and flour. The application of the different dry heat treatments as pre-processing methods is reviewed, followed by the effect of these treatments on starch chemistry, structure and functionality as tested in dry heat-treated flour and flour produced from heat-treated wheat kernels.

## 2. Dry Heat Treatment

Dry heat treatment of wheat kernels and flour is considered a convenient, safe and reliable technique to achieve physical changes of the starch properties [19]. Dry heat treatment, normally used as a pre-processing method, results in the reduction of the moisture content of the treated sample. The extent of the moisture loss is determined by the severity of the treatment, which is related to the exposure time, temperature and pressure. In addition, the nature of the raw material contributes to the effectiveness of the treatment [4]. In the case of whole wheat, the initial moisture content, size of the grain, structure, chemical composition and shape influence the final moisture content of the heat-treated samples [20]. 

Bucsella et al. [18] reported changes in the functional properties of wheat flour after dry heat treatment due to changes in protein-related properties. Compared to bread wheat flour, cake flour showed more significant improvements after the treatment [18]. Cakes produced from dry heat-treated flours have shown enhanced dough stability, stronger foam structure and increased volume [2,6,18]. Roasting is usually a high-temperature short-time dry heat treatment process where mass, moisture and heat transfer takes place [10]. When used as a heat treatment for whole wheat, the kernels may puff due to the increased vapour pressure and the expansion of the internal structure [21].

During dry heat treatment, processing conditions, water activity as well as the addition of heat enable interactions between the wheat components (amino acids and reducing sugars), resulting in the occurrence of the Maillard reaction. In addition, caramelisation takes place which allows for the development of improved colour, aroma and flavour as well as increased antioxidant activity [12,22]. At high temperatures, detrimental compounds such as acrylamide, a carcinogenic compound formed during the Maillard reaction between asparagine and a reducing sugar, can occur [23]. Acrylamide formation can be limited by reducing the treatment time and temperature. This could, however, reduce the desired components formed during the Maillard reaction. Hence, it is necessary to optimise the process conditions, such as temperature and time, that will reduce the formation of such detrimental compounds while enhancing the sensory properties of the processed kernel.

Dry heat treatment of whole wheat kernels or flour involves heat transfer through convection, conduction or radiation. Roasting is achieved by using sand, a fluidising bed roaster, a conventional oven or a forced convection continuous tumble roaster (FCCT). Radiation methods include microwave heating and infrared radiation whereas gun puffing, vacuum steam and superheated steam thermal methods are based on a change in pressure. 

### 2.1. Roasting

#### 2.1.1. Forced Convection Continuous Tumble (FCCT) Roasting

**Figure 1:**
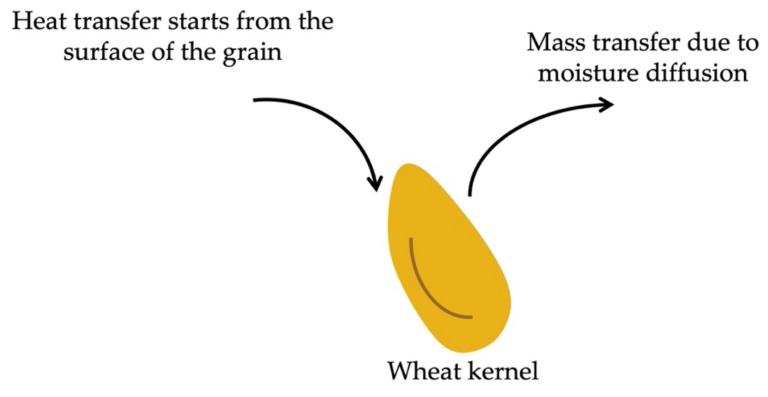
Schematic of convection dry heat treatment of wheat kernels during force convection continuous tumble (FCCT) roasting by means of heat and mass (moisture diffusion) transfer.

The principle of forced convection has been known for a long time, but the use of forced convection continuous tumble (FCCT) roasting in cereal products has only emerged in recent years. It is based on the principle of using forced airflow at atmospheric pressure to roast a sample. Hot air is continuously circulated throughout the chamber, allowing for even heat transfer and increased efficiency [24]. The rotating mixer at the centre of the chamber allows for products to move through the chamber during the roasting process [11,24]. FCCT uses dry air as a heat transfer medium. However, dry air can be replaced with heated steam by pre-roasting a test sample that has been tempered to 18–20% moisture content. The moisture from the test sample will evaporate as semi-superheated steam and form a part of the heating medium, allowing for more efficient and even heat transfer [11]. Heat transfer starts from the surface of the wheat kernel and progresses inwards as the duration of the treatment increases (Figure 1). Therefore, changes in the starch properties commence from the grain surface. However, at higher temperatures, starch granules near the surface of the grain will undergo more severe changes, i.e., complete gelatinisation and pasting. Mass transfer in the form of moisture diffusion also commences from the surface of the grain. FCCT roasting allows for even roasting on all surfaces of the product, the efficient use of energy and accurate temperature control [21,25,26]. Further advantages include that this roasting method allows for simple working principles, it has multiple product applications and it requires minimal maintenance [11]. Previous studies have shown that the FCCT roasting of wheat results in increased antioxidant activity at an optimal high temperature and time combination [26], as well as maintaining the protein quality and protein content at optimal conditions (90 °C; 130 s), which are required to produce high-quality breads [24]. Compared to conventional oven roasting at a similar temperature and time combinations, FCCT allows for less detrimental changes in the structural, physicochemical and functional properties of flour milled from roasted wheat [11]. However, at extreme treatment conditions (180 °C; 140 s), moisture losses, denaturation of proteins, thermal decay of polysaccharides as well as increased kernel porosity and lowered relative density were observed [11].

#### 2.1.2. Sand Roasting

**Figure 2:**
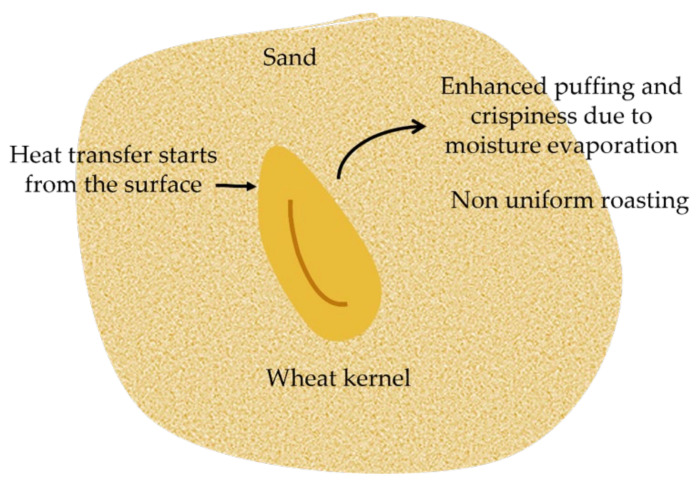
Schematic of mode of heat and mass transfer during conduction sand roasting.

Sand roasting is a traditional method used in India for grain processing [27]. It involves the use of a steel pan heated by liquid petroleum gas and sand as a heat transfer medium. The temperature and time combinations of the wheat kernel roasting conditions in a study by Gujral et al. [27] ranged from 250 to 350 °C for 15–100 s. Enhanced puffing and crispiness, due to mass transfer in the form of moisture diffusion (Figure 2), was observed in the roasted kernel at these high temperatures. No puffing was observed in a more recent study by Dhua et al. [28] when the wheat kernels were roasted at a lower temperature of 175 °C for 60 s. The water and oil absorption capacity of the milled flour increased, whereas the emulsifying, foaming capacity and pasting properties decreased. This method, however, produces non-uniformly roasted kernels with a high silica and acid-insoluble ash content due to sand contamination [10]. Furthermore, due to conduction heating with sand as the main heat transfer method, the outer areas of the sample can become over-roasted whilst the core remains untreated [28].

#### 2.1.3. Fluidised Bed Roaster (FBR) 

**Figure 3:**
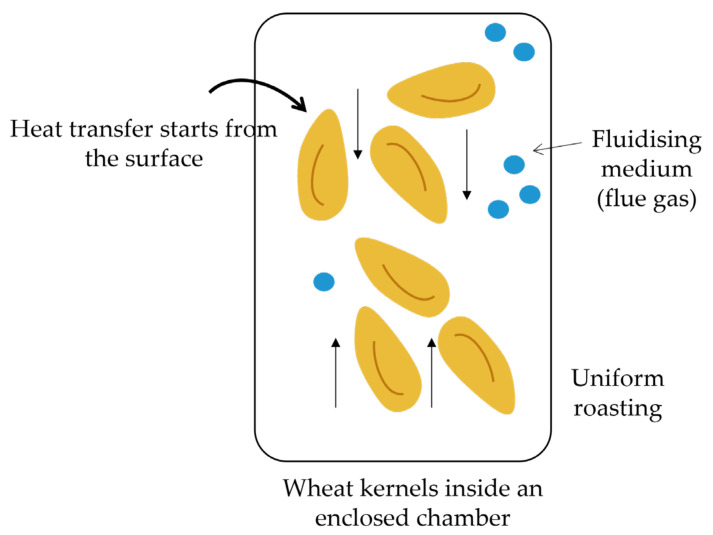
Schematic overview of mode of heat transfer during convection heating using a fluidised bed roaster (FBR)—the wheat kernels are exposed to a fluidised medium (i.e., flue gas).

The fluidised bed roaster (FBR) provides a roasting technique in which a solid sample is exposed to a fluidising medium such as flue gas, allowing the solid sample to act as a liquid (Figure 3). A vibratory feeder is used to transfer samples at a constant rate through the roasting, resting and cooling decks. An electric motor is used to control the frequency and amplitude of vibration [10]. Temperature and time combinations ranging from 280 to 350 °C for 40–50 s allow for optimal high-temperature roasting. However, to achieve homogeneous roasting, the treatment conditions should be adjusted according to the size and density of the kernels [10]. The wheat kernels move at a high speed during the roasting to uniformly expose all surfaces and allow for even heat and mass transfer throughout the sample. As a result, moisture is lost. This technique is advantageous, as maintenance costs are low, the equipment has a simple design and the grains are homogeneously roasted without mechanical damage to the sample. Despite the improved colour and the reduction in the energy required to mill the roasted wheat, its effect on the functional properties of the starch in the wheat flour has not been determined to date. 

### 2.2. Radiation

#### 2.2.1. Microwave Heating 

**Figure 4:**
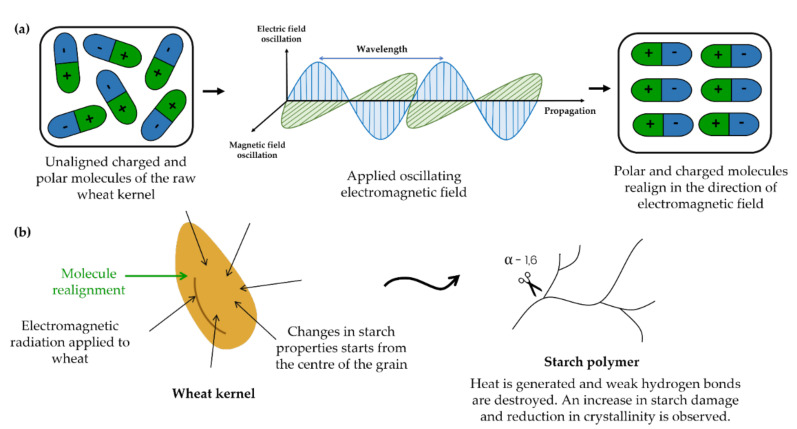
Schematic representation of the mechanism of electromagnetic radiation as applied to wheat kernels. (**a**) Realignment of charged and polar molecules in the direction of the applied field and (**b**) changes in the wheat kernel starting from the centre of the grain.

Dry heat treatment by means of microwave heating is advantageous, as it has the ability to achieve high heating rates, the start-up time is short, the energy is efficiently used and little space is required [29]. Microwave heating of food samples occurs by means of the absorption of microwave radiation and its conversion into heat (Figure 4). Previous studies have investigated the microwave treatment of wheat kernels and wheat starch and its effect on the functional and physicochemical properties of the flour and starch [7,29]. A study by Padalino et al. [7] involved the hydration of wheat kernels to 19–21% moisture content, drying at ambient temperature to remove excess exterior water (17–20% moisture content) and heat treatment in a microwave oven for 5 min at 600 W. This resulted in early disulphide bond formation in the polymeric proteins during the heat treatment, preventing the later interaction with the starch molecules during the processing of the wheat flour, as well as preventing the formation of a gluten network. Subsequently, it affected the pasting properties of the wheat flour, where higher water absorption values and swelling indexes were recorded. However, increased cooking losses in the final products, such as pasta, was observed due to the weak gluten network formation.

Dhua et al. [28] noticed a decrease in the pasting properties as well as a significant reduction in the emulsifying capacity of the flour produced from the microwave-treated wheat kernels (5 min; 600 W). This may be ascribed to protein denaturation and surface hydrophobicity after the heat treatment. The heat treatment used by Palav and Seetharaman [29] involved a solution of wheat starch and distilled water, placed in a cylinder and covered to prevent moisture losses, and heating in a microwave oven (4300 W) for 10, 20 and 30 s. The microwave power absorbed during the treatment was 1140 W. In contrast to the study by Padalino et al. [7], Palav and Seetharaman [29] observed a lack of granule swelling. This may be ascribed to the solitary presence of the starch in the latter study, whereas the weak gluten network formed in the first study allowed for the granules to expand and absorb more moisture. Rapid temperature increase during the microwave heating resulted in a weaker gluten protein configuration, increased starch granule rupturing, the absence of a continuous amylose network and a reduction in swelling power [28,29]. 

#### 2.2.2. Infrared (IR) Treatment 

Infrared (IR) radiation provides an efficient heat treatment—the processing time is short, as the temperature of the sample rapidly increases during IR heating [20]. IR radiation is that part of the electromagnetic spectrum with wavelengths longer than visible light but shorter than those of microwaves [30]. As heat transfer occurs through electromagnetic waves (Figure 4), this method relies on the ability of a sample to absorb electromagnetic radiation. IR radiation increases the internal energy that generates molecular vibrations, resulting in a change in the dipole moment. A photon with the same energy is emitted, which ultimately generates heat. 

Using IR radiation as a dry heat treatment increased the digestibility of the starch and appeared to be effective in decontaminating low moisture foods from bacteria, yeasts and moulds [20]. Wheat kernels were heated with four IR radiators whilst travelling on a conveyer belt. The processing temperature was determined by the grain surface temperature and the time ranged from 100 to 180 °C and 30 to 150 s, respectively. The treatment severity, with respect to the temperature, was altered by changing the distance between the conveyer belt and the radiator. A reduction in the grain compressive resistance was observed, as the treatment severity increased due to the damage of the internal grain structure. 

Radiation penetrates the grain, giving rise to heating that begins at the centre of the grain [20]. Heat is transferred through conduction towards the outer layers until an equilibrium temperature is reached. The extent of the radial penetration is dependent on the wavelength, where longer wavelengths result in less penetration. Changes in the starch structure were also observed from the centre of the grain, supporting the fact that radiation penetrates the grain and heating starts from the centre.

### 2.3. Pressure

#### 2.3.1. Superheated Steam Treatment

**Figure 5:**
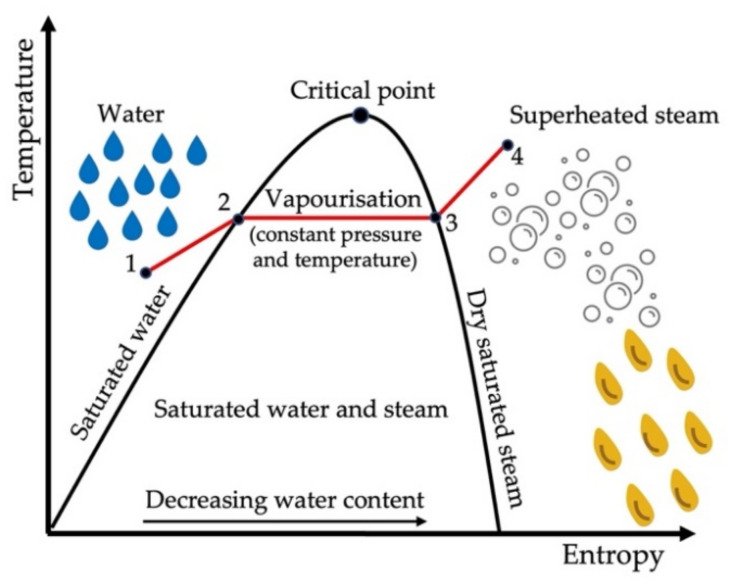
Schematic diagram of converting water into superheated steam to be used as dry heat treatment of whole wheat kernels. Water is heated to boiling point (1 to 2) after which it is vapourised at constant pressure (2 to 3) and dry steam is superheated (3 to 4).

In recent years, the use of superheated steam (SS) on wheat has emerged as a viable pre-processing method to enhance product quality. It uses sensible heat to increase the temperature of the product above its saturation temperature at a specified pressure (Figure 5). Steam will not condense for as long as the temperature is kept above saturation conditions [31]. It has become a viable processing technique to modify the functional properties of starch-based products as well as to dry foodstuffs, owing to the high heat transfer coefficient during processing [16]. SS is created by reducing the pressure of the saturated steam [31]. An oxygen-free environment is created, reducing the oxidative degradation and ensuring that no combustion reactions occur [2,31]. When a product is exposed to SS, heat is transferred when the steam condenses on the grain surface. Consequently, a small amount of moisture is gained. Once the product temperature reaches the saturation temperature, the moisture will evaporate and the product drying will commence [2]. The evaporated moisture is recycled to recover the energy within the system [31].

SS drying is associated with higher temperatures and shorter time duration compared to conventional drying. This can mitigate the losses of heat-sensitive compounds [12]. Previous studies have shown that SS processing heats the food while retaining the vitamins and other essential nutrients [31]. However, when lower temperatures and shorter time are used, the sample will absorb the condensed steam, resulting in harder and more compact kernels [2]. This induces additional damaged starch during milling. Furthermore, slight protein denaturation and starch gelatinisation occurs, which makes the wheat flour suitable for products such as cakes, noodles and biscuits [6]. The study of Liu et al. [32] concluded that treatment conditions at 190 °C for 1 min of soft wheat flour was sufficient to improve the specific volume, texture and sensory qualities of cake—possibly due to the increased batter viscosity and decreased batter density. 

#### 2.3.2. Vacuum Steam Treatment 

Vacuum steam treatment is a promising pre-processing technique for low-moisture food products such as wheat to inactivate the microbial load, owing to its high level of heat transfer [8]. Vacuum steam treatment allows for heat treatment under 100 °C with the use of a vacuum [33]. Snelling et al. [8] showed that using this treatment at 65 °C reduced the microbial load while maintaining the protein quality and functionality of wheat flour. However, under severe conditions, the gelatinisation of starch and increased water absorption indexes occurred. A sample was pre-treated with forced hot air at 40 °C to ensure equal temperatures throughout the sample. Subsequently, the sample was sealed in a basket with ports available for steam addition and vacuum application, as well as measurements for temperature and pressure. To maintain maximum steam conditions within the basket, the pressure should be adjusted according to the processing temperatures. The sample was kept within the treatment environment at temperatures ranging from 65 to 85 °C and pressures from 230 to 627 mbar for the selected time. As the processing increased the moisture content to 16%, the sample was dried at room temperature overnight or until the moisture content dropped below 14% [8,33]. Even though this method calls for low-temperature processing, which lowers the costs, this method can be time consuming, as overnight drying is required. 

#### 2.3.3. Gun Puffing 

Gun puffing is based on instantaneous evaporation and expansion when the pressure of the wheat grains containing superheated steam is reduced to a low pressure [22,34]. The grains are often further processed and can ultimately be used in products such as breakfast cereals and ready-to-eat snack foods, owing to their pre-gelatinised starch, crispiness and lightness. Mariotti et al. [34] heated wheat kernels between 105 and 115 °C for 90 s followed by puffing at 1.3–1.5 MPa for 75–85 s. This resulted in an expected increase in the kernel size and a drastic change to the ultrastructure of the grains. A substantial increase in damaged starch was observed with a subsequent increase in the water absorption capacity. Most of the starch granules were completely gelatinised during treatment. In contrast, Cattaneo et al. [22] exposed wheat kernels to heated air at 360–390 °C for 3–5 min, after which steam was injected into the puffing chamber between 7 and 10 bar for 1 to 4 min. Finally, the chamber was opened and the immediate drop in pressure led to the vaporisation of moisture and expansion of the grain. These high temperatures maximised the expansion and resulted in notable levels of the Maillard products hydroxymethylfurfural and furfural. 

## 3. Properties of Starch in Heat-Treated Wheat 

### 3.1. Chemical and Structural Properties

#### 3.1.1. Chemical Composition of Starch Granules 

Starch is comprised of two glucose polymers, namely amylose and amylopectin, that influence its physicochemical properties [35]. Heat treatment leads to changes within the amylopectin structure, as amylopectin is more heat sensitive. This is attributed to the steric hindrance of branching—α-1,6 glycosidic bonds are much smaller than the linear α-1,4 glycosidic bonds and, as a consequence, the α-1,6 linkages are more easily broken [19]. Heat treatment as a pre-processing step disrupts the molecular structure of the starch and reduces its molecular weight due to the fragmentation and depolymerisation of the high molecular weight amylopectin into shorter chains with a lower molecular weight [19]. This increases the accessibility for enzymatic hydrolysis [5,36]. 

α-Amylase and β-amylase digest amylose and amylopectin to produce maltodextrins consisting of D-glucose units with different chain lengths [37]. Dry heat treatment resulted in a weaker gel and a lower viscosity was formed, affecting the functional properties of the starch [4]. Quick hydrolysis is only feasible if the moisture content is sufficient and the temperature is suitable for α-amylase activity, as the hydrolysis rate is temperature-dependent. At increased temperatures, α-amylase activity can be promoted or the thermal stability may be jeopardised, inactivating the enzymes [2,8,37]. Hu et al. [2] noticed a reduction in α-amylase activity after SS treatment at 110–170 °C. However, no significant reduction occurred during the vacuum steam treatment [8]. On the contrary, the α-amylase activity increased during the FCCT roasting at 180 °C for 140 s [11]. In a high-temperature environment, reducing sugars, e.g., maltose, interacts with proteins, resulting in the formation of melanoidins on the outside surface as a consequence of the Maillard reaction [23].

#### 3.1.2. Endosperm and Starch Granule Morphology 

The morphology of wheat starch granules greatly contributes to the functional properties of wheat flour. Schoeman and Manley [11] compared the effect of oven roasting with FCCT roasting on the ultrastructure of the endosperm of wheat kernels. Oven-roasted kernels had an expanded structure with open pores, increased porosity, volume and lowered density. Increased porosity was attributed to the internal moisture diffusion and moisture loss during roasting at high temperatures (180 °C; 140 s). Large visible cracks on the surface of the endosperm were also observed in oven roasting. Conversely, less destructive changes in the internal structure and even roasting occurred after the FCCT roasting [11]. While intermediate SS treatment resulted in a slight expansion of the wheat endosperm, mild treatment resulted in a compact structure due to absorbed moisture [2]. Eroded starch granule surfaces with dents were also observed during the dry heating [19].

Sand roasting and microwave heating resulted in a lowered bulk density after the heat treatment [38]. The destruction of the starch–starch or starch–protein interactions, or the increased voids within the starchy endosperm, can be a plausible reason for this phenomenon. The non-uniform heat transfer during the sand roasting resulted in a denser structure compared to the FBR, which displayed an open, less compact structure [10]. Andrejko et al. [20] also noted internal structural changes after IR radiation, where cracks were observed in the centre of the grain which progressed outwards as the processing time and temperatures increased. The compression resistance of the wheat grains exposed to IR radiation also decreased. As the treatment severity increased, lower compression force values were observed.

Mariotti et al. [34] noticed significant changes in the internal structure of the starch granules after gun puffing. Noticeably, gun puffing did not only result in cavities forming within the starch granules, but partially ‘fused’ starch granules and proteins were also observed. A non-homogenous endosperm structure was therefore observed, with certain areas highly compacted while others represented large cavities. The excessive moisture losses and expansion during gun puffing resulted in wheat kernels with a lower bulk density due to lower weight and larger puffed volume [34]. Partial gelatinisation and protein denaturation due to the SS thermal treatment gave rise to the formation of starch–starch and starch–protein clusters, increasing the particle size distribution [16].

#### 3.1.3. Crystallinity 

The crystallinity of wheat starch is determined by the ratio of amylose and amylopectin, the number of double helices, the extent of amylopectin destruction as well as the crystal size [19,39]. Higher amylose content usually indicates a lower crystallinity, as amylopectin is the main crystalline component of starch [19]. The crystallinity patterns, i.e., A-, B-, C- and V-type polymorphs, determine the molecular characterisation of starch [40,41]. A-type crystallites are predominant in cereal starch as it contains only eight water molecules between each double helix, which are tightly packed within each monoclinic crystal unit [42]. V-type crystalline structures occur in cereal starches, indicating co-crystallisation of the single helix amylose with other complexes such as lipids, proteins, fibres, alcohols or other small polar molecules [41]. Heat treatment can induce the formation of V-type crystallites in wheat starch, along with facilitating the realignment of linear chain amylose to form double helices, and therefore increasing the degree of crystallinity [40]. However, severe heat treatment results in the destruction of the molecular order, decreasing the crystallinity of starch [43].

The proportion of A- and B-type granules in the bimodal distribution of wheat starch affects the structural and functional properties of wheat [44] and, evidently, the quality of the end product. The spatial arrangement of the crystalline regions, rather than the extent of the crystallinity, determines the accessibility of the enzymes to reach the binding sites within the granule [45]. Moreover, it was shown that heat treatment disrupted the crystalline order of native wheat starch and its realignment upon cooling promoted the formation of new crystallites [42]. A reduction in the relative crystallinity of starch granules due to the increase in temperature during the SS treatment of wheat flour has also been demonstrated [16,43]. This could be attributed to the destruction of the hydrogen bonds from the double helices, damaging the crystalline region [16]. V-type crystallites were also observed after the SS treatment, indicating the interaction between the starch molecules and the polar organic molecules or the formation of amylose–lipid complexes [16,43].

V-type polymorphs are divided into Type I and Type II complexes depending on the thermal transitions and the degree of crystallinity of the produced complex. High-temperature treatment (>90 °C) of low moisture foods resulted in the formation of Type II amylose–lipid complexes forming clear semi-crystalline structures [46]. Oven and FCCT roasting of wheat kernels did not significantly affect the crystallinity [11], possibly due to the low moisture content and short roasting time (140 s). Microwave treatment promotes the vibration of water molecules within the crystalline regions of the starch granule, resulting in heat generation and the molecular order destruction of amylopectin. Consequently, the starch granules lost their birefringence at much lower temperatures than the gelatinisation temperature, as demonstrated by Palav and Seetharaman [29]. Similar observations occurred after the IR heating; however, the melting of the crystallites started from the centre of the grain and progressed outwards [20]. 

#### 3.1.4. Milling Performance and Starch Damage

Milling of wheat affects the structural and functional properties of flour (Table 1), such as the granular morphology and the molecular weight, the thermal and pasting behaviours as well as the water absorption ability and solubility [47]. This results in flours with different physicochemical properties. Certain milling methods such as hammer milling and stone milling involve grinding the whole kernel into flour [47,48]. The size of the endosperm, bran and germ is reduced. However, it is accompanied by physical damage to the endosperm, as small fragments are broken away from the starch granule [47,49]. The friction created during milling causes the grain to heat up, and moisture is lost [47,48].

The extent of the starch damage content is not only related to the milling technique used but also to the hardness of the wheat. Wheat is classified as soft, medium or hard, depending on the endosperm texture and the protein content [48]. Milling of hard wheats results in the increase of damaged starch, as it is more difficult to mill the tightly packed protein–starch matrix within the endosperm into small particles. Smaller particle size usually relates to a greater amount of starch damage [2]. In contrast, the endosperm of soft wheats is less densely packed due to the weaker starch–protein interactions [50]. Since the hardness of the wheat used in thermal treatment studies is often not considered, it is not always possible to explain the extent of the damaged starch observed.

The type and severity of the heat treatment determine the extent of the starch damage. Snelling et al. [8] noticed no significant changes in starch damage content after the vacuum steam treatment of wheat kernels. Intermediate SS temperatures ranging from 140 to 170 °C resulted in a less tightly packed endosperm that was easier to break [2]. Moisture evaporation and expansion during thermal treatment may be ascribed to the loosely packed endosperm [11]. Compared to the untreated wheat samples, less starch damage occurred during the milling of the intermediate SS-treated kernels [2]. However, the mild processing treatment, i.e., 110 °C for 1 min, resulted in kernels absorbing condensed steam and developing a denser structure due to the stronger bond between the starch granules and the proteins. Mechanical damage in this starch structure arose during milling and resulted in increased damage to the starch as the breakage occurred at the endosperm cell walls [51]. Furthermore, other thermal treatment processes also resulted in more highly damaged starch content compared to the control samples. The FCCT roasting of wheat kernels at 180 °C for 140 s resulted in a significant increase in starch damage content, which is attributed to the vast amount of moisture losses during roasting [50]. Another plausible reason can be ascribed to the insufficient moisture absorption during the tempering of the roasted wheat kernels prior to milling, resulting in the fragmentation of the starchy endosperm during flour production [50]. 

The change in pressure and the remarkably high processing temperatures used during gun puffing resulted in kernels with nearly complete disintegration of the starch granules. Values of up to 80% of damaged starch or gelatinised starch were observed as a result [34]. Excessive starch damage (>10%) is detrimental during breadmaking, as it will lead to a poor final loaf quality [5,47].

### 3.2. Functional Properties

#### 3.2.1. Thermal Behaviour of Starch

Starch gelatinisation, requiring excess water and heat, involves the absorption of water, resulting in several molecular transitions, i.e., granule swelling, the loss of birefringence, the reduction of crystallinity and the disruption of the granule structure [23]. In a limited water environment and in the presence of heat (e.g., dry heat treatment), the intermolecular hydrogen bonds within the starch molecule are broken down—the starch will, however, only be partially gelatinised [3,4,16]. The technique and severity of the treatment involved determine the thermal behaviour of the wheat starch, as described in Section 3.2.2. Increasingly damaged starch may occur during milling due to mechanical forces [47,49]. The number of adsorption sites, for α-amylase, on the starch surface is determined by its accessibility and porosity [37]. Damaged starch exposes more binding sites on the disrupted granule, increasing the susceptibility of enzymatic hydrolysis and, therefore, water absorption [5,35,36,52]. Upon further heating in the presence of water, the starch granule eventually ruptures, allowing for soluble amylose molecules to leach out into the external water phase, and a starch paste is formed. The addition of shear forces allows for the total disruption of the granule [23,39].

#### 3.2.2. Degree of Gelatinisation 

Moisture plays an essential role during the gelatinisation of starch molecules. Pores and cavities on the starch surface allow for enhanced moisture migration into the granule [39]. Ma et al. [16] found, during heating in limited water, that starch will partially gelatinise; however, the degree of gelatinisation will increase with increasing treatment temperatures.

Gelatinisation enthalpy, i.e., the energy required for a sample to gelatinise, indicates the melting of double helices and the disruption of the molecular order [53]. Thus, gelatinisation enthalpy is dependent on the moisture content during treatment, the processing temperature as well as the intactness of the double helices present in the crystalline and amorphous regions of the starch granules [11]. A-type granules contribute to higher gelatinisation enthalpy values and pasting temperatures, as they contain a greater amount of ordered short-chain structures. B-type granules have a greater amount of amylose–lipid complexes, resulting in lower gelatinisation enthalpy, broader gelatinisation ranges and increased swelling power [26]. Schoeman and Manley [11] indicated that, irrespective of granule type, a partial pre-gelatinised starch will exhibit decreased gelatinisation enthalpy due to the loss of its molecular structure, and therefore an increased gelatinisation temperature range.

Granule size also influences the degree of gelatinisation. Larger granules demonstrated higher gelatinisation enthalpy, indicating a more ordered arrangement of polysaccharide chains, thus a greater crystallinity [54]. This is supported by the fact that small starch granules have lower crystallinity, which increases their affinity to bind water. Therefore, less energy is required to induce gelatinisation [54]. Oven-roasted samples showed a higher degree of gelatinisation due to the increased disruption of the molecular order compared to the FCCT roasting [11]. Processing temperatures exceeding 150 °C for longer than 90 s during the IR radiation resulted in a decrease in the apparent grain strength and the damage of the internal kernel structure due to the onset of starch gelatinisation [20]. Heating through electromagnetic waves allows for the starch to absorb heat and gelatinise from the centre of the grain, proceeding outwards. 

#### 3.2.3. Water Absorption

Water absorption is dependent on the presence of hydrophilic groups to bind water molecules [11,28]. Partial gelatinisation allows for strengthened interactions between the water and polar groups from the exposed starch granules, consequently enhancing the ability of the granules to bind and retain water [16]. Denatured proteins also have an increased accessibility of polar groups due to protein unfolding, enhancing the capability of retaining water molecules within its structure [11]. A small amount of starch damage enhances the ability of flour to absorb water, allowing for a gel to be easily formed [49]. Wheat samples treated with SS and vacuum steam (higher than 85 °C) were partially gelatinised, and improved water absorption indexes were observed [8,16]. However, temperatures lower than 75 °C during the vacuum steam treatment resulted in a decrease in water absorption [8]. Oven roasting resulted in a higher water absorption capacity compared to FCCT roasting, as more destructive changes and large voids in the endosperm occurred [11]. Furthermore, the hardness of the wheat comes into play again. Soft wheats are milled into small, finer particles, which enhance water diffusion and swelling. The opposite is seen in hard wheats, as milled particles are coarse [11]. 

Padalino et al. [7] indicated that the microwave treatment (600 W; 5 min) of wheat kernels prior to milling affected the arrangement of polymeric proteins. Covalent disulphide bonds between gluten proteins formed during the heat treatment, which prevented the formation of a strong gluten network during pasta production. This enabled starch granules to absorb more water during cooking, which decreased the hardness of the product. In addition, the leaching of amylose to the surface promoted increased cooking losses [7]. Dhua et al. [28] explained that the effect of microwave heating on protein–starch and starch–starch interactions changed the functional properties of the flour, which may be associated with the rapid water removal. Palav and Seetharaman [29], on the other hand, showed that the protein–starch interactions did not contribute to functional property changes in microwave treated wheat starch. The starch granules ruptured, preventing the formation of strong gels, and consequently a lack of granule swelling was observed. The fast-heating rate and vibrational motion of the water molecules prevent the starch granules from undergoing the normal gelatinisation process during the slow heating in water, i.e., water absorption, swelling, the loss of birefringence and amylose leaching. Instead, the pressure generated during the heat treatment results in the expansion of the granule. The rate of hydration is therefore much slower than the rate of expansion. Consequently, the strain on the granule structure results in the collapse of the starch granule. 

Similar outcomes occur in wheat kernels exposed to gun puffing. The highly expanded structure due to cavity formation, the porosity of the internal structure and the structural changes of the outer layers of the puffed grains led to a much greater ability to absorb water. The puffed wheat kernels can hydrate four to five times more water than raw grains within the same period [34]. 

#### 3.2.4. Solubility of Starch and Swelling Power

In a limited moisture environment at high temperatures, starch and protein will compete for moisture absorbance. Starch has the ability to gelatinise, and the protein will form a gluten network [55]. Thermal treatment denatures the protein and disrupts the hydrogen bonds linking the starch molecules together, resulting in partial gelatinisation. It has been shown that SS treatment of wheat flour results in the formation of a protein matrix and a partially gelatinised starch layer forms a barrier around the intact starch granules [16]. The protein barrier could retard water uptake. In the presence of an aqueous phase, clusters of the agglomerated structures such as protein–starch, starch–starch or starch–lipid aggregates limit the water uptake and diffusion of amylose as well as the soluble molecules. This results in a decreased solubility index of starch. In other words, denatured proteins can restrict the water solubility index. Furthermore, the rearrangement and strengthening of the molecular order during thermal treatment are also responsible for a decline in solubility [6].

It is known that amylose may retard the swelling of starch granules by stabilising the granule structure. Therefore, amylopectin (and even more so for long-chain amylopectin molecules) greatly contributes to the water absorption, swelling ability and pasting behaviour of starch, which explains why waxy starches (amylose-free granules) have great swelling power [56]. However, water-holding capacity, hence, swelling power, is greater in flour milled from wheat that experienced thermal treatment through SS [6] and roasting [11]. This may be attributed to the weakening of the internal matrix of starch granules, enhancing interactions between water and flour.

Mariotti et al. [34] showed that during gun puffing the aleurone layer of the gelatinised endosperm was not destroyed and remained in the puffed wheat grain. Certain macromolecules within the aleurone layer, such as fibres, may be partially depolymerised. As a result, the depolymerised molecules are more soluble in water, and the water solubility index of puffed wheat grains was greater than raw wheat grains.

#### 3.2.5. Pasting

Pasting profiles are influenced by changes in the functional properties of wheat flour, such as the degree of gelatinisation, complex formation with other compounds and α-amylase activity. The changes in viscosity, measured by a Rapid Visco Analyser (RVA), are recorded as a function of time and temperature to determine the pasting behaviour of a starch sample [39]. Thermal treatment of wheat flour by SS resulted in wider pasting temperature ranges, decreased pasting temperatures and higher viscosities [57]. The minimum temperature required to cook flour is presented by the pasting temperature [39]. Thermal treatment decreased the pasting temperature of flours, and with further increase in temperature or time, even lower pasting temperatures were observed [2]. This can be explained by the decline in the crystallinity of the starch granules observed during the thermal treatment due to breakage of intermolecular hydrogen bonds and the unfolding of double helices, resulting in a greater proportion of amorphous regions. 

Peak viscosity correlates with the resistance of swollen granules to shear, which relates to the swelling power of starch [6]. It greatly contributes to the texture and quality of final products. Schoeman and Manley [11] stated that higher peak viscosity indicates a greater degree of starch gelatinisation. Increased peak viscosity occurs through starch resisting the shear forces due to protein cross-linking and enhanced interactions between the denatured proteins and starch granules [4,6]. Furthermore, higher viscosities, including peak, trough and final viscosity, could be attributed to the changes in proteins, allowing for the greater ability to absorb and retain water [4,6,43]. These changes include the formation of a more ordered protein structure due to the modification in hydrophobicity [4] or the denaturation of proteins surrounding the starch granules [16]. SS treatment also decreased the α-amylase activity and starch damage content of flours, resulting in less destruction at the inner regions of the starch molecular chains and thus higher pasting profiles and higher viscosities [2,4,37,43]. Similar viscosity and pasting temperature changes occurred after the FCCT and oven roasting of wheat kernels; however, these changes were not significant [11]. Even though changes in the structural composition and functional properties occurred during the dry heat treatment, the obtained results may be attributed to the increase in the α-amylase activity after roasting. The latter would result in a decreased viscosity, as it hydrolyses bonds within the inner regions of the starch structure [37]. 

Extensive SS heat treatment promoted the melting and gelatinisation of starch, which decreased the viscosity of the flour due to the inability of the starch granules to absorb water and swell [2,6]. This is in accordance with a study by Mariotti et al. [34] showing that gun puffing led to a substantial amount of damaged starch and the increased capacity of the flour to absorb water. Moreover, a lower and less pronounced peak viscosity was observed, as most starch granules were already completely gelatinised [34]. 

During microwave cooking, the vibrational motion of the water molecules and the re-orientation of the other polar molecules to the oscillating microwave field greatly affected the pasting properties of the wheat flour [7,29]. As microwave cooking has a very high heating rate, the kinetic limitation restricts the absorption of water and the swelling of the starch granules. Consequently, the molecular vibrations disrupt the hydrogen bonds within the granule, resulting in amylose spilling into the medium in the form of molten polymers rather than swelling and leaching [29]. These starch granules lose their birefringence at much lower temperatures compared to granules undergoing other heat treatments. It has also been reported that the amount of reducing sugars produced by α-amylase during the microwave treatment was low. Therefore, less granule disintegration occurred [29]. All these factors are supported by the fact that microwave cooking results in starch granules with lower pasting temperatures due to the low-temperature gelatinisation, the higher peak viscosities due to the incomplete destruction of the granule integrity and the lower final viscosity due to less amylose leaching.

The hardness of the wheat, which relates to the protein content, once again leads to changes in the functional properties. The pasting profile of the flour produced from the wheat kernels with a higher protein content was shown to be different from those containing less protein [18]. A significant increase in the peak viscosity was observed when the high-protein-content flour underwent dry heat treatment due to structural changes within gluten proteins. FCCT-roasted soft wheat kernels milled into flour had increased viscosities since a larger exposed surface area from the finer particles contributed to more water being absorbed [11]. The opposite was observed for flours produced from roasted hard wheat kernels, as the particles were rougher, limiting water uptake and starch swelling. Studies have shown that wheat-based products such as cakes and noodles produced from milled heat-treated kernels will result in higher viscosities and therefore improved mouthfeel and texture [2,32].

#### 3.2.6. Cooling of Starch Gels

Upon the cooling of the non-crystalline starch structures, amylose and amylopectin molecules reassociate to form a new crystalline order. Retrogradation, therefore, involves the transition of a rubbery state starch solution to an ordered crystalline structure in the form of a viscoelastic gel during cooling [23,39]. The crystalline pattern is dependent on the moisture content and the level of gelatinisation, as the mobility of the amylose and amylopectin chains are limited in an ordered structure [3]. Association of amylose is predominantly responsible for short-term changes, as the linear regions easily aggregate through hydrogen bonding, whereas the recrystallisation of amylopectin is responsible for long term changes in final products [58]. However, factors such as the amylose–amylopectin ratio, the water content, temperature, the presence of lipids, sugars and hydrocolloids as well as the chemical and physical modifications extensively affect the rate of retrogradation [3].

Breakdown viscosity indicates the heating stability of a cooked gel, where its value relates to the rate of rupture of swollen starch granules when maintained at a constant high temperature [39]. FCCT and oven roasting of wheat kernels resulted in flour with an increased breakdown viscosity compared to the control sample, indicating an increase in the rate of starch granule rupturing [11]. Starch granules with a low tendency to retrograde will display lower setback viscosities [39]. FCCT and oven roasting resulted in a decrease in setback viscosity compared to the control sample [11]. The increase in peak viscosity, possibly due to additional α-amylase activity after roasting, may be attributed to the increase in the breakdown and reduction in setback values. However, in samples treated with SS where a reduction in α-amylase activity was observed, the breakdown, as well as setback viscosity, increased. The increase in viscosity and gel formation upon cooling is ascribed to the association of amylose [28]. Dhua et al. [28] and Palav and Seetharaman [29] noticed lowered final viscosity values after microwave heating and the sand roasting treatment. Gun puffing resulted in a limited viscosity increase upon cooling, as the starch was already completely gelatinised during the thermal treatment [34]. Therefore, the partial or complete gelatinisation of starch granules is responsible for the reduced gel stability [2,6,28].

With regards to end-product quality, moisture greatly contributes to bread staling, as the moisture migration from the gluten and starch matrix within the crumb to crust are responsible for crumb firmness [58]. Hu et al. [43] observed that heat treatment led to increased water uptake and water retention, which positively impacts bread quality to a certain extent. As a result, decreased bread firmness and enhanced crumb softening was observed. Palav and Seetharaman [29] noticed less disruption of the molecular order during microwave heating, and a lack of granule swelling occurred, the interaction of amylose molecules during storage resulted in the absence of a continuous network to be formed and a soft gel was created. The level of firmness increased with an increase in treatment time and the solid content of the starch gel [29]. Padalino et al. [7] also reported that the formation of a weak gluten network during microwave heating enhanced the absorption of water, which reduced the firmness of the cooked sample.

Vacuum steam treatment at 85 °C for 4 and 8 min showed a significant increase in the firmness of the crumb texture [8]. However, at lower treatment temperatures, the increase in the firmness was not significant [8]. This is attributed to the amount of damaged starch granules, the degree of gelatinisation and the rate of retrogradation. High crystallinity relates to low initial firmness [59]. 

During baking, higher temperatures result in more retrogradation [58]. This corresponds to the findings of Fu et al. [3], who reported that highly disordered structures would retrograde faster than ordered structures. A more ordered structure reduces the mobility of starch macromolecules, which delays double helix formation during retrogradation [3]. One can therefore argue that partial gelatinisation before baking, which relates to the low degree of crystallinity and the disruption of the molecular order, is responsible for the increased firmness of the crumb texture due to rapid retrogradation. In addition, the retrogradation of amylopectin is greatly responsible for the crumb firmness during storage, as it entraps water molecules within its crystallites, resulting in the dehydration of the crumb matrix [58].

#### 3.2.7. Interactions with Other Components 

Thermal treatment disrupts the starch granule structure and induces protein aggregation. As a result, protein–starch crosslinking, amylose–lipid complexes and inter- and intramolecular hydrogen bonding occur, which evidently forms an uninterrupted structure increasing the diameter of the granule [16]. Protein greatly contributes to changes in the behaviour of starch. SS treatment at temperatures ranging from 130 to 170 °C was used to evaluate the effect of the modified wheat flour on cake quality. Denatured proteins surrounded the partially gelatinised starch granules and delayed further gelatinisation of the starch during cooking. Increased gelatinisation temperature, viscosity and swelling power were observed, which positively affected the quality of the cake [6]. Therefore, flour modification is beneficial in the production of cakes. In contrast, this negatively affects the quality of bread, as SS weakens the gluten strength of dough and causes the starch to have an inability to properly bind to gluten [5,6]. Dhua et al. [28] noticed an increase in the oil absorption capacity after sand roasting and microwave cooking due to the dissociation of proteins and the increase in non-polar binding sites, allowing for the interaction with the hydrocarbon tail.

Schoeman and Manley [11] noted a decrease in protein content during roasting at 180 °C. Germishuys et al. [60] also indicated that FCCT roasting, at relatively high temperatures (between 130 and 150 °C), resulted in flours of which a dough could not be formed due to less protein–protein interactions. Therefore, high-temperature heat treatment will denature the protein, resulting in a weak gluten strength due to the inability to form a gluten network [16]. However, at 90 °C for 130 s, Germishuys and Manley [24] noticed no significant differences in the protein content, and improved bread foam properties were observed. This could potentially be due to an increase in dough strength as a result of protein polymerisation and the development of a stronger gluten network through protein and starch interactions [61].

Non-polar lipids are found within the germ and aleurone layer. However, during milling, approximately half of these lipids are transferred to the produced flour. Upon storage, lipase hydrolyses triacylglycerols into free fatty acids, monoacylglycerol and diacylglycerol, which result in acid rancidity. Acid rancidity negatively affects the baking quality of whole wheat flour by reducing its functional properties [62]. Fortunately, whole wheat flour contains antioxidant-rich bran, and heat treatment through roasting increases the bioavailability of antioxidants that slows down the enzymatic activity during storage [12,13,62]. It has been reported that the microwave heating of wheat kernels, bran and whole wheat flour effectively minimises lipase, lipoxygenase, polyphenol oxidase and peroxidase activities [63]. Polyphenol oxidase activity, however, did not significantly decrease during the vacuum steam treatment [8]. Lipase naturally occurs in the bran and not in the endosperm [62]. Even though lipase is more heat stable than peroxidase, the enzymatic activity decreased as the heat treatment severity increased. Excessive heat treatment can also denature the antioxidants that would have maintained the oxidative stability [62]. The high amount of unsaturated fatty acids together with lipase and lipoxygenase deteriorate the wheat germ oil upon storage due to the formation of volatile compounds [64]. Gili et al. [64] found that IR radiation of 4800 W/m^2^ for 3 min at a 0.2 m emitter–sample distance was the optimal condition to stabilise the wheat germ without significantly reducing the antioxidant content. 

Amylose–lipid complexes are naturally occurring structures often formed during heat treatment. As lipids are hydrophobic, these complexes have an inability to absorb water, consequently affecting starch functional properties, including pasting, swelling power, the leaching of amylose, viscosity and the retrogradation of starch [45,65]. Furthermore, starch–protein interactions, or the protein barrier surrounding starch, prevents the availability of enzyme binding sites on the starch granules [16]. It should be noted that the number of binding sites on starch fractions increases due to structural changes during thermal treatment. The digestibility of starch is therefore influenced, as it is determined by the potential of enzymes to bind starch molecules.

## 4. Conclusions 

Dry heat treatment of wheat generally results in the partial gelatinisation of starch due to modifications in its structural properties. In addition, a reduction in damaged starch occurs after the milling of wheat kernels previously exposed to intermediate heat treatment conditions. However, mild and severe treatments generate an increase in starch damage content. Thermal treatment of wheat and/or flour also increases the amorphous content, resulting in changes in the functional behaviours of starch, e.g., increasing its viscosity and lowering the pasting temperature. The decrease in α-amylase activity observed during thermal treatment may also contribute to an increase in viscosity. In contrast, FCCT and oven roasting also increased the α-amylase activity but increased (insignificantly) viscosity was observed. Similar trends in the changes in the functional and structural properties of starch occurred due to dry heat treatment. New interactions, due to the heat treatment, of starch with proteins, lipids or other starch molecules may result in a lowered solubility index but an enhanced ability to retain water. Heat treatment, at optimal conditions, is a viable solution for the pre-processing of wheat flour to enhance its utilisation. Temperature, time and tempering conditions appear to be very important variables in influencing the quality of the resulting products from heat pre-processed wheat kernels. Future studies are therefore required to optimise these conditions to produce flour with better functionality and improved utilisation.

## Figures and Tables

**Table 1:** Dry heat treatment methods and impact on structural and functional properties of starch in wheat flour compared to control samples.

Dry Heat Treatment Methods	Structural and Functional Properties	References
Sand roasting	Significant puffing; lowered bulk density Increased water absorption capacity	[38] [28]
Microwave heating	Lower pasting temperatures; higher peak viscosity; lower final viscosity; lack of granule swelling; increased water absorption during cooking	[7,29]
Infrared treatment	Reduction of grain compressive resistance; gelatinisation from the centre of the grain	[20]
Gun puffing	Significant puffing; lower peak and final viscosity; increased water absorption capacity and water solubility indexes	[34]
Vacuum steam treatment	No change in functionality at optimum conditions	[8]
Superheated steam	Partial gelatinisation; wider pasting temperature range; decreased pasting temperature; increased viscosity; higher swelling power; increased breakdown and setback viscosity	[2,43]
Forced convection continuous tumble (FCCT) roasting	Gelatinisation; increased water absorption capacity; increased pasting properties; less destructive changes and less gelatinisation compared to oven roasting	[11]
